# Supplementary material for: The Circadian Rhythm of Intracellular Protoporphyrin IX Accumulation Through Heme Synthesis Pathway in Bladder Urothelial Cancer Cells Exposed to 5-Aminolevulinic Acid
Source: Cancers (Basel). 2024 Dec 8;16(23):4112. doi: 10.3390/cancers16234112 (PMC11640531; doi:10.3390/cancers16234112)
Supplement: Supplementary file 1 [file cancers-16-04112-s001.zip › cancers-3278849-supplementary.pdf]

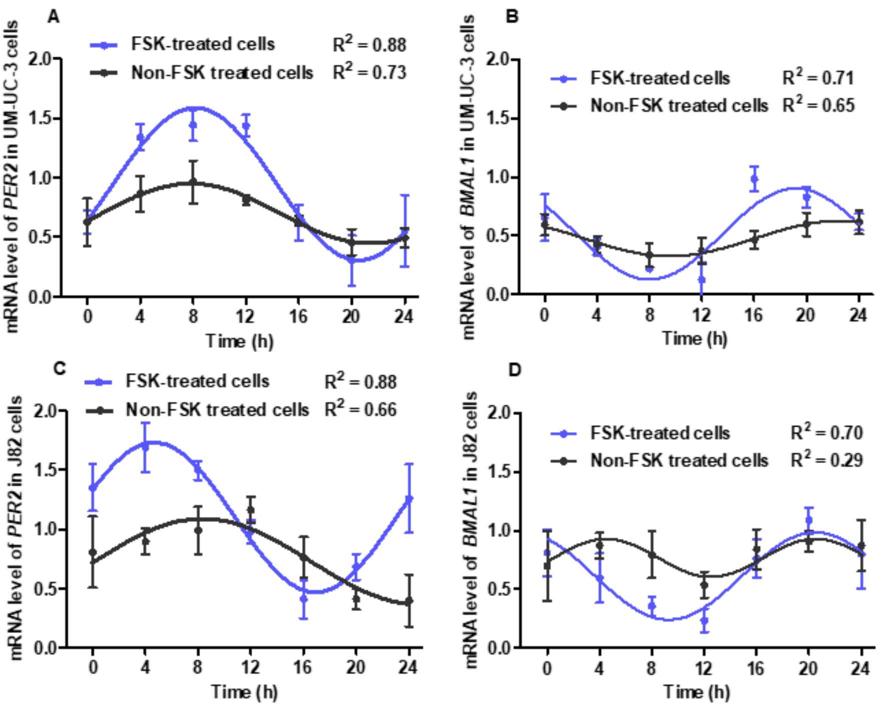

Supplementary Materials: Figure S1: Two bladder urothelial cancer cell lines, UM-UC-3 and J82, were exposed to FSK (10 µM in 0.5% FBS). Two hours after FSK exposure, mRNA levels of *PER2* and *BMAL1* were measured every 4 hours during 24 hours in both the FSK-exposed group and the control group. Figures A and B show the results for UM-UC-3 cells, and Figures C and D show the results for J82 cells.
